# Serum amyloid A is a potential predictor of prognosis in acute ischemic stroke patients after intravenous thrombolysis

**DOI:** 10.3389/fneur.2023.1219604

**Published:** 2023-07-06

**Authors:** Qi Chang, Yaqiang Li, Min Xue, Chuanqing Yu, Jiale He, Xun Duan

**Affiliations:** ^1^Department of Neurology, First Affiliated Hospital of Anhui University of Science and Technology (First People’s Hospital of Huainan), Huainan, China; ^2^Department of Neurology, People’s Hospital of Lixin County, Bozhou, China

**Keywords:** inflammation, acute ischemic stroke, serum amyloid A, C-reactive protein, intravenous thrombolysis

## Abstract

**Objectives:**

Inflammation shows a notable relationship to acute ischemic stroke’s (AIS) occurrence and prognosis. However, existing research has confirmed that serum amyloid A (SAA) is an inflammatory biomarker. The aim of this paper was to investigate the association between SAA and the three-month clinical results of acute AIS patients after intravenous thrombolysis (IVT).

**Methods:**

The evaluation of AIS patients with complete medical records was carried out by prospectively investigating patients hospitalized in our department between January 2020 and February 2023. The SAA levels were examined with the use of an immunosorbent assay kit that shows a relationship with the enzyme (Invitrogen Corp). Patients were dichotomized into favorable (mRS score of 0, 1 or 2) and unfavorable (mRS score of 3, 4, 5, or 6) results with the use of the modified Rankin Scale (mRS).

**Results:**

A total of 405 AIS patients who were subjected to IVT therapy were prospectively covered. To be specific, 121 (29.88%) patients had an unfavorable prognosis during the follow-up for 3 months. On that basis, patients achieving unfavorable results gained notably greater SAA levels (39.77 (IQR 38.32–46.23) vs.31.23 (IQR 27.44–34.47), *p* < 0.001) during hospitalization in comparison to patients with a better result. In the analysis with multiple variates, SAA was adopted to achieve the independent prediction of the three-month unfavorable clinical results of acute AIS patients after IVT [OR:2.874 (95% CI, 1.764–4.321), *p* < 0.001]. When the fundamental confounding factors were regulated, the odds ratio (OR) of unfavorable prognosis after AIS patients undergoing IVT therapy was 4.127 (95% CI = 1.695–10.464, *p* = 0.032) for the maximum tertile of SAA in terms of the minimal tertile. With an AUC of 0.703 (95% CI, 0.649–0.757), SAA revealed a notably more effective discriminating capability in terms of CRP, NLR, EMR, and WBC. SAA as a predictor in terms of the prediction of three-month unfavorable results after AIS patients undergoing IVT therapy achieved specificity and sensitivity of 84.45% and 77.23%, as well as an optimal cut-off value (COV) of 37.39.

**Conclusion:**

SAA level that is up-regulated during hospitalization is capable of serving as an effective marker in terms of the prediction of unfavorable three-month results in AIS patients after IVT.

## 1. Introduction

Acute cerebral infarction (ACI) is characterized by high mortality, disability, and morbidity rates. The existence of an ischemic hemispheric zone after ACI has been confirmed through continuous clinical research such that the treatment of this disease has been leaping forward (1, 2). In general, ACI is attributed to arterial blockage by a thrombus, triggering limited tolerance of the brain tissue to ischemia. With the prolongation and intensification of ischemia, the necrotic area of the ischemic center tends to expand such that the ischemic semi-dark zone can be reduced (3). Nevertheless, the study demonstrated that although emergency intravenous thrombolysis (IVT) is capable of carrying out rapid and early restoration of blood supply to the brain, shortening the time of ischemic damage, reducing the infarct area, and improving neurological damage, post-thrombolysis thrombolytic complications (e.g., intracranial hemorrhage, revascularization, and ischemia–reperfusion injury) may occur and affect the prognosis of patients (4).

Ischemic stroke destabilizes the balance existing based on the quiescent state of coagulation and immunization axes within the brain, resulting in ischemic lesions’ local inflammation and a peripheral immunization response (5–8). Directly inducing neuronal cell death arising from inflammatory responses, oxidative stress, and blood-brain barrier disruption are all considered to be significant pathogenic processes in acute ischemic stroke (AIS) (9, 10). Several studies have investigated the relationship between serum inflammatory markers and functional results in AIS patients (11–13). A high neutrophil-to-lymphocyte ratio (NLR) during hospitalization is a marker that is effective in terms of the prediction of unfavorable short-term results in mild AIS patients after IVT (14). The level of high neutrophil counts to the ratio of lipoprotein cholesterol at a high density (NHR) also showed a relationship to unfavorable three-month results after IVT among patients subjected to AIS (15). In addition, an increased systemic inflammatory response index (SIRI) as well as mean platelet volume to lymphocyte ratio (MPVLR), and decreased eosinophil to monocyte ratio (EMR), especially SIRI during hospitalization, showed a significant relationship with unfavorable clinical results of mild AIS after IVT (16–18).

Serum amyloid protein A (SAA) refers to an acute time-responsive protein that affects the transport and clearance of cholesterol by altering the function of high-density lipoprotein (HDL) such that lipid deposition can be triggered, and the development of atherosclerosis and atherosclerotic vascular disease can be accelerated (19, 20). SAA is also an apolipoprotein, which is present in the serum’s HDL fraction and takes on critical significance in the chemotaxis of inflammatory cells to inflammatory sites (21). Moreover, its synthesis is primarily achieved through hepatocytes; it is an extensively employed non-specific clinical marker of inflammation, taking on critical significance in diagnosing acute and chronic inflammation. SAA is a useful biomarker for diagnostic confirmation of atherothrombotic ischemic stroke (22). Furthermore, a study carried out recently has confirmed that greater levels of serum SAA, showing a notable relationship to hemorrhagic severity and inflammation, developed an independent relationship with unfavorable results after aneurysmal subarachnoid hemorrhage (23). Additionally, research revealed that risen SAA shows a notable relationship with conditions of inflammation (e.g., myocardial infarction, intracerebral hemorrhage, post-stroke cognitive impairment, and atherosclerosis) (24–27).

Recently, existing research confirmed that SAA is capable of predicting several diseases’ prognoses (e.g., ischemic stroke receiving endovascular thrombectomy, severe coronavirus disease, and advanced pancreatic cancer) (28–30). So far, SAA has been rarely explored as neurological results’ prognosis marker. Besides, a cutoff SAA in terms of the prediction of unfavorable results in AIS patients undergoing IVT therapy has been scarcely proposed. Accordingly, this study had the aim of carrying out a systematic investigation of SAA’s relationship with functional results among AIS patients subjected to IVT therapy. Furthermore, a cutoff SAA value was built as a marker of prognosis in terms of the evaluation of functional results in AIS patients undergoing IVT therapy.

## 2. Materials and methods

### 2.1. Subjects

**Figure 1:**
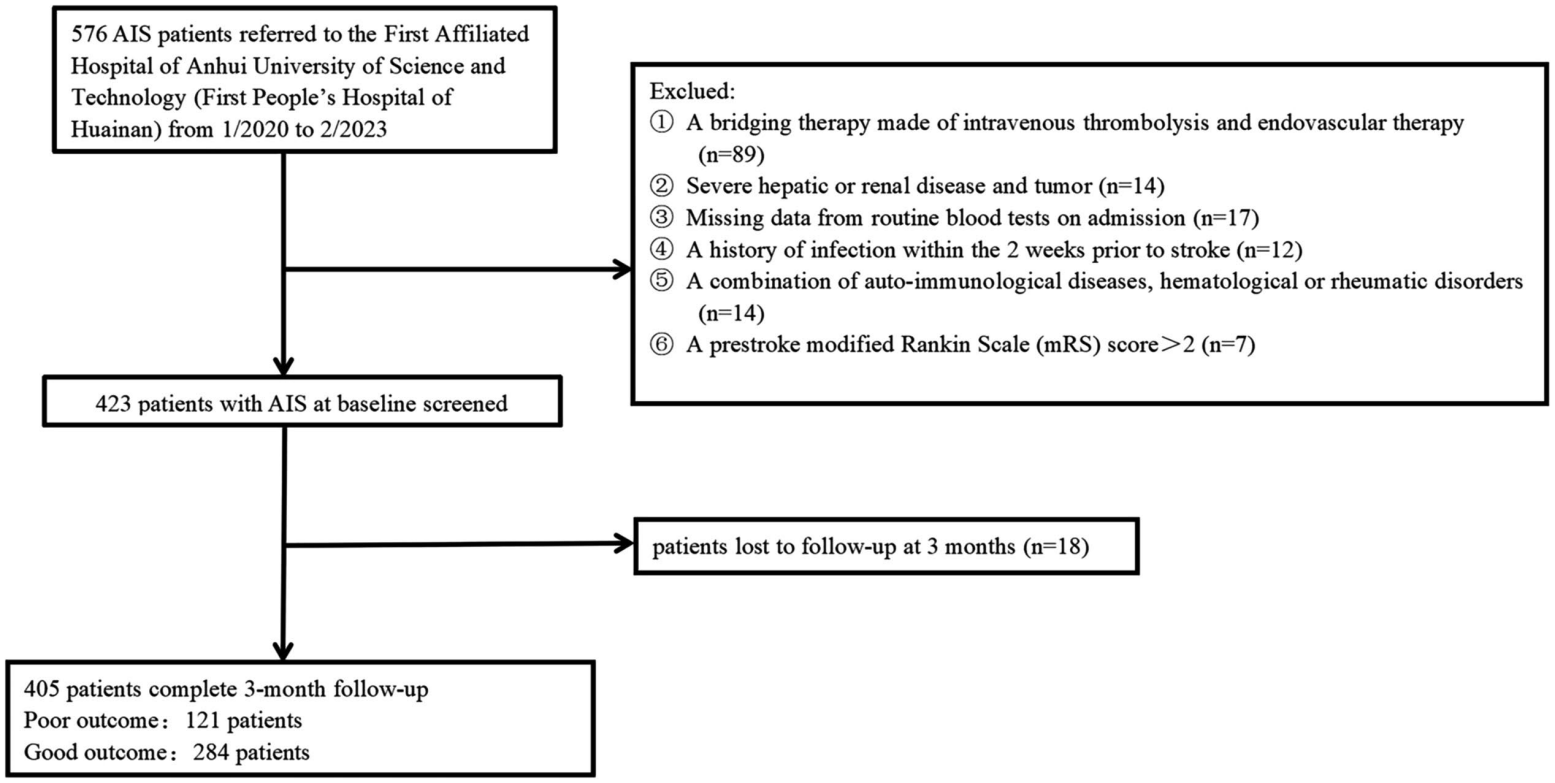
Flowchart of participant selection.

All patients who had consecutive follow-ups following AIS diagnosis and administration using intravenous rt-PA in 4.5 h stroke onset or intravenous urokinase in 6 h stroke onset in Anhui University of Science and Technology’s First Affiliated Hospital (First People’s Hospital of Huainan) from January 2019 to February 2023 have been recruited to the present prospective observational analysis. Patients were not covered if they: (1) were subjected to a bridging therapy made of intravenous thrombolysis and endovascular therapy; (2) had missing baseline data, three-month mRS scores, and NIHISS scores; (3) achieved a pre-stroke modified Rankin Scale (mRS) score >2; (4) had a combination of other serious diseases, for instance, recently severe infections, severe liver and kidney diseases, auto-immunological diseases, hematological or rheumatic disorders, and malignant tumors; (5) had an onset-to-treatment time (OTT) of more than 6 h; (6) had no follow-up visit or missed visit. Finally, the study covered a total of 405 AIS patients (Figure 1). The study gained approval from the participating center’s institutional ethics review board (approval number: 2022-KY-207-001). Patients signed informed consent following the 1975 Declaration of Helsinki.

### 2.2. Data collection and assessment

Patients’ demographic data (marital state, education level, gender, and age), vascular risk factors (alcohol consumption, atrial fibrillation, diabetes mellitus, history of hypertension, and smoking status), and lesion location were obtained from the electronic medical records. To evaluate the stroke severity during hospitalization and 3 months after hospitalization, we adopted the National Institutes of Health Stroke Scale (NIHSS). The Barthel Index (BI) and the Mini-Mental State Examination (MMSE) were employed for the evaluation of functional results and cognitive function, separately, after a 90-day follow-up. Additionally, the primary endpoint followed the prognostic assessment 3 months after the admission, which was performed at 3 months by two trained neurological physicians unaware of the patients’ results in the laboratory. Clinical visits or telephone interviews after 3 months of AIS can determine patients’ clinical results, with the use of the modified Rankin Scale (mRS). Patients were dichotomized into favorable (mRS score of 0, 1, or 2) and unfavorable (mRS score of 3, 4, 5, or 6) results based on mRS. In addition, the variables regarding clinical results were collected, including time to needle initiation (ONT), Alberta Stroke Program Early CT Score (ASPECT), infarct volume, Trial of Org 10,172 in Acute Stroke Treatment (TOAST), and door-to-needle time (DNT) (31). On-admission CT imaging of early ischemic variations was evaluated with ASPECTS. ASPECTS were scored ranging from 10 (no early signs of ischemia) to 0 (early ischemic variations in all 10 regions). For the respective identified region, 1 point was subtracted from 10.

### 2.3. Blood collection and laboratory test

Patients’ blood samples were collected from peripheral venous blood on an emergency basis prior to IVT. The conventional laboratory methods were performed to measure the apolipoprotein B (ApoB), apolipoprotein A (ApoA), low-density lipoprotein-cholesterol (LDL-C), high-density lipoprotein cholesterol (HDL-C), triglycerides (TG), total cholesterol (TC), C-reactive protein (CRP), serum glucose (G), lymphocyte (L), neutrophil (N), monocyte (M), eosinophil (E), platelet (Plt) count, white blood cell count (WBC), and homocysteine (Hcy). The ratio of neutrophil count to lymphocyte count, eosinophil count to monocyte count, and platelet count to lymphocyte count are expressed as NLR, EMR, and PLR, separately. With the use of an enzyme-linked immunosorbent assay kit (EHSAA1, Invitrogen, Carlsbad, California, United States), operated rigorously following the guideline of the producer, the SAA level was examined. The sensitivity of the detection of serum SAA by ELISA was 500 pg./mL. For ELISA, the intra-and inter-assay coefficients of variation (CV) were 10% and 12%, respectively. This ELISA kit showed no cross-reactivity with other soluble structural analogs. To minimize detection errors and improve detection accuracy, a professional clinical technician with no knowledge of clinical results or neuroimaging results was responsible for the random analysis of all samples on the identical day.

### 2.4. Statistical analysis

Statistical investigation was carried out with the use of SPSS for Windows (version 22.0, Inc., Chicago, IL, United States). Continuous variables with normal distribution have the expression of means (e.g., standard deviations), variables with non-normal distribution have the expression of medium value and interquartile range (IQR), and categorical variables have the expression of percentages and counts. The Kolmogorov–Smirnov test was performed to evaluate the distributions’ normality. The optimal cutoff value (COV) for SAA was obtained with the use of Receiver operating characteristic (ROC) analysis in terms of the prediction of clinical results in AIS patients after IVT. We then calculated the AUC on SAA to measure the accuracy of the test. Continuous data with normal distribution were compared using the *t*-test, non-normally distributed continuous variables were compared using the Mann-Whitney U-test, and categorical variables were compared between groups using the relevant Fisher exact or Pearson χ2 tests. The differences among the three groups as well as between the two groups when testing for abnormal distributions for continuous variables were compared based on the Mann–Whitney U test and the Kruskal-Wallis test. Pearson or Spearman rank correlation analysis was conducted for the analysis of the bivariate correlations. Logistic regression analysis was carried out based on multiple variates for identifying risk factors that achieved the independent prediction of unfavorable results after 3 months of thrombolytic therapy for AIS. The admission SAA was considered for dividing patients into tertiles (T1 ≤ 29.23, T2 29.33–35.44, T3 ≥ 35.48). We performed multivariate regression analysis by applying three models to identify factors capable of predicting unfavorable results, with model 1 aiming at marital status, education level, sex, and age; model 2 aiming at model 1 as well as vascular risk factors (alcohol consumption, atrial fibrillation, diabetes mellitus, history of hypertension, and current smoking); model 3 targeting variables with *p* < 0.05 demonstrated based on the univariate analysis (WBC, CRP, EMR, NLR, WBC, CRP, small vessel occlusion, cardioembolism, basal ganglia, three-month BI score, ASPECT score, infarct volume, NIHSS score during hospitalization, and three-month NIHSS score). Furthermore, the relationship has an expression of OR with 95% CI. *p* < 0.05 indicated statistical significance.

## 3. Results

### 3.1. Baseline characteristics exhibited by patients in the unfavorable result and favorable result groups

**Figure 2:**
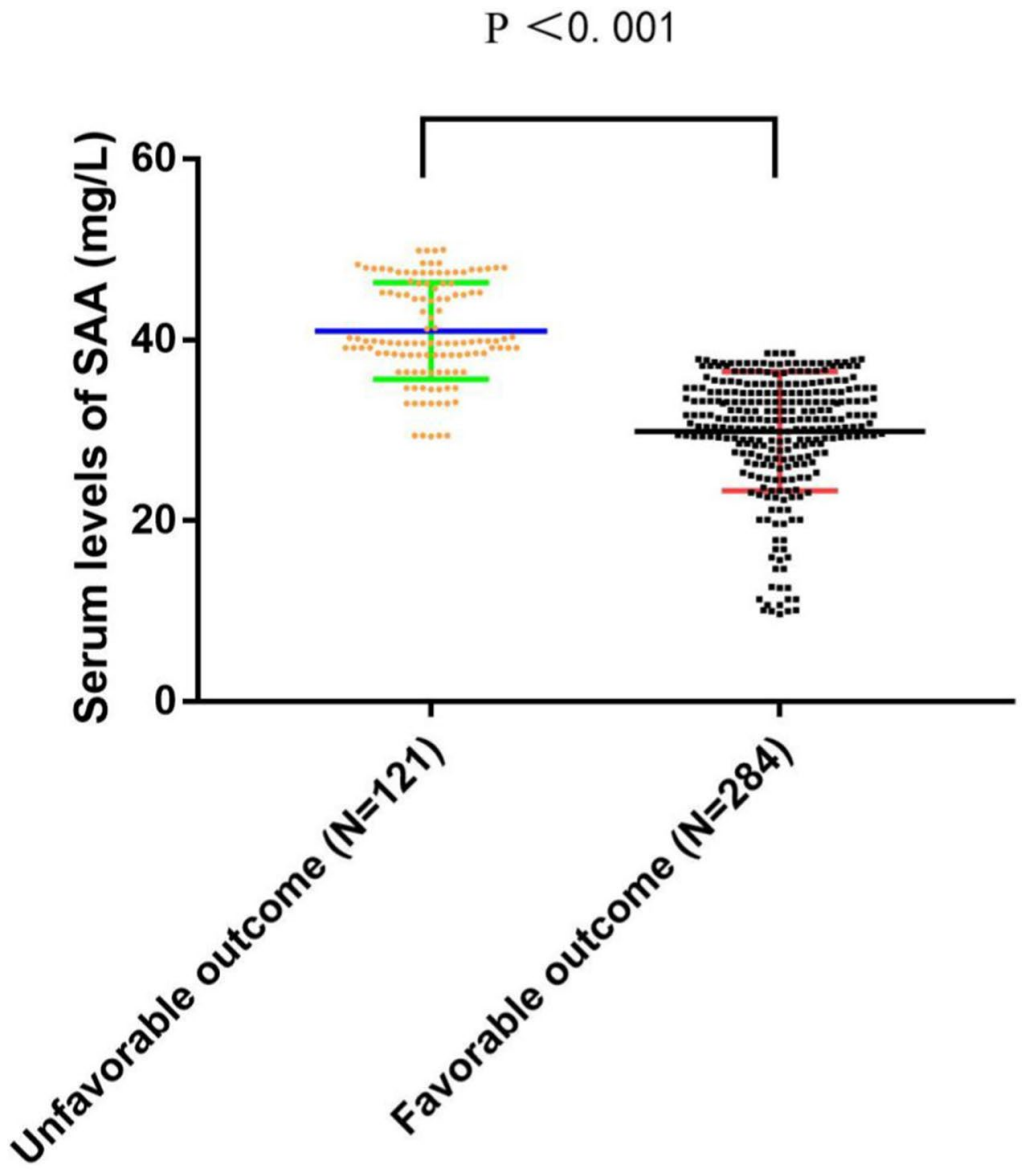
The comparison of SAA levels between the unfavorable results and favorable results. SAA denotes serum amyloid A. SAA levels are the medium values and interquartile ranges.

AIS patients were enrolled according to participating centers between January 2020 and February 2023. We first recruited 487 patients, followed by excluding 82 patients, including 18 patients who were not followed up at 3 months, 17 patients who had missing data on routine blood tests during hospitalization, and 136 patients complying with the exclusion standards (e.g., a bridging therapy made of intravenous thrombolysis and endovascular therapy, a history of infection within the 2 weeks prior to the stroke, severe hepatic or renal disease and tumor, a combination of auto-immunological diseases, hematological or rheumatic disorders, and a pre-stroke modified Rankin Scale (mRS) score >2). Finally, the study yielded 405 patients (224 women, aged 66.08 ± 8.11 years), which covered 121 (29.88%) patients in the unfavorable result group after AIS and 284 (70.12%) patients in the favorable result group after AIS. Table 1 presents the comparison of the baseline characteristics between the two groups of patients. In terms of patients in the favorable result group after AIS, those in the unfavorable result group presented greater NIHSS scores during hospitalization (*p* < 0.001), greater three-month NIHSS score (*p* < 0.001), greater WBC (*p* < 0.001), greater CRP (*p* < 0.001), greater SAA (*p* < 0.001), greater ASPECT score (*p* < 0.001), greater infarct volume (*p* < 0.001), lower EMR (*p* = 0.028), greater NLR (*p* = 0.048), greater proportions of cardioembolism (*p =* 0.001), lower proportions of small vessel occlusion (*p =* 0.002), and lower BI score (*p* < 0.001). Among lesion locations, the basal ganglia (*p* = 0.014) showed a notable relationship with the unfavorable result risk. Figure 2 demonstrates that serum SAA levels were notably greater in patients with an unfavorable prognosis of AIS than in the favorable prognosis group.

**Table 1:** Clinical and demographic characteristics of patients in favorable and unfavorable results groups.

Variables	Total (*n* = 405)	Three-month functional result		*p* value
		Unfavorable result (*n* = 121)	Favorable result (*n* = 284)	
**Demographic characteristics**				
Gender, female, *n* (%)	224 (55.31)	66 (54.55)	158 (55.63)	0.318
Age, years, mean ± SD	66.08 ± 8.11	66.01 ± 8.09	66.23 ± 8.18	0.805
Education years, medium value (IQR)	5 (3–7)	5 (3–7)	5 (3–8)	0.137
Married, *n* (%)	356 (87.90)	98 (80.99)	258 (90.85)	0.073
Vascular risk factors (%)				
Hypertension	287 (75.80)	91 (75.21)	216 (76.06)	0.855
Diabetes mellitus	193 (47.65)	52 (42.97)	141 (49.65)	0.218
Coronary heart disease	65 (16.05)	24 (19.83)	41 (14.44)	0.176
Atrial fibrillation	53 (13.09)	18 (14.88)	35 (12.32)	0.486
Current smoking	157 (38.77)	44 (36.36)	113 (39.79)	0.517
Alcohol consumption	123 (30.37)	36 (29.75)	87 (30.63)	0.860
ONT, min, mean ± SD	162.73 ± 47.45	161.94 ± 47.60	163.07 ± 47.46	0.826
DNT, min, mean ± SD	67.95 ± 23.45	64.67 ± 21.18	69.35 ± 24.26	0.092
ASPECT, medium value (IQR)	10 (9–10)	9 (8–10)	10 (9–10)	**<0.001**
Infarct volume, ml, medium value (IQR)	4.43 (1.36–20.42)	21.51 (3.99–66.79)	2.68 (0.92–12.65)	**<0.001**
**Laboratory findings (IQR)**				
Plt, × 10^9^/L, medium value (IQR)	214 (177–247)	216 (158–261)	214 (182–241)	0.973
SAA, mg/L, medium value (IQR)	33.23 (29.28–37.66)	39.77 (38.32–46.23)	31.23 (27.44–34.47)	<0.001
WBC, ×10^9^/L, medium value (IQR)	6.07 (5.26–7.29)	7.0 (5.77–8.52)	5.94 (5.22–6.81)	<0.001
CRP, mg/L, medium value (IQR)	6.61 (5.70–7.66)	7.44 (6.25–8.53)	6.3 (5.58–7.38)	<0.001
EMR, mean ± SD	0.58 ± 0.62	0.49 ± 0.39	0.62 ± 0.69	0.028
PLR, mean ± SD	170.64 ± 40.52	185.51 ± 35.52	165.05 ± 36.63	0.051
NLR, mean ± SD	3.59 ± 3.26	4.28 ± 0.95	3.29 ± 1.70	0.048
Glucose, mmol/L, medium value (IQR)	5.30 (4.70–6.85)	5.30 (4.70–6.70)	5.30 (4.70–6.90)	0.815
TG, mmol/L, medium value (IQR)	1.36 (0.95–1.95)	1.29 (0.98–1.83)	1.39 (0.94–2.02)	0.206
TC, mmol/L, medium value (IQR)	4.50 (3.77–5.39)	4.53 (3.71–5.22)	4.47 (3.77–5.45)	0.943
HDL-C, mmol/L, medium value (IQR)	1.02 (0.85–1.24)	1.03 (0.86–1.27)	1.02 (0.85–1.21)	0.503
LDL-C, mmol/L, medium value (IQR)	2.55 (1.93–3.17)	2.58 (2.00–3.15)	2.55 (1.92–3.20)	0.442
ApoA, g/L, medium value (IQR)	1.26 (1.13–1.43)	1.26 (1.13–1.43)	1.27 (1.09–1.44)	0.819
ApoB, g/L, medium value (IQR)	0.86 (0.69–1.03)	0.86 (0.69–1.05)	0.85 (0.69–1.02)	0.662
Hcy, μmol/L, medium value (IQR)	12.89 (10.36–16.31)	13.02 (10.82–16.73)	12.66 (10.19–15.93)	0.249
Lesion location, *n* (%)				
Frontal lobe	66 (15.07)	16 (13.22)	49 (17.25)	0.312
Parietal lobe	49 (12.10)	13 (10.75)	36 (12.68)	0.585
Temporal lobe	114 (28.15)	32 (26.45)	82 (28.87)	0.619
Occipital lobe	55 (13.58)	14 (11.83)	41 (14.78)	0.441
Basal ganglia	196 (48.40)	71 (58.68)	125 (44.01)	0.007
Brainstem	31 (7.65)	10 (8.26)	21 (7.39)	0.763
Cerebellum	46 (11.36)	18 (14.87)	28 (9.86)	0.145
**TOAST subtype (*n*.%)**				
Large artery atherosclerosis	160 (39.51)	41 (33.88)	119 (41.90)	0.131
Cardioembolism	116 (28.64)	48 (39.67)	68 (23.94)	0.001
Small vessel occlusion	69 (17.04)	11 (9.09)	58 (20.42)	0.005
Other or undetermined	60 (14.81)	21 (17.36)	39 (13.73)	0.348
**Neuropsychological function**				
NIHSS score during hospitalization, medium value (IQR)	6 (4–8)	10 (9–13)	5 (4–6)	<0.001
Three-month NIHSS score, medium value (IQR)	3 (1–5)	6 (4–8)	2 (1–3)	<0.001
MMSE score, medium value (IQR)	22 (18–26)	23 (17–25)	22 (18–26)	0.918
Three-month BI score, medium value (IQR)	2 (2–3)	4 (3–4)	2 (2–2)	<0.001
Three-month mRS score, medium value (IQR)	80 (60–90)	50 (40–65)	90 (70–90)	<0.001

*** *p* < 0.001.

### 3.2. Baseline characteristics exhibited by all patients in SAA tertiles

SAA level tertiles were considered to divide all patients into three subdivided groups, ensuring that each subdivided group had sufficient patient types from 2.87 to 51.98 (T1, 135 patients; T2, 135 patients; T3, 135 patients). The cut-off values (COV) for stratifying the SAA into tertiles were: (T1) 2.45–29.23, (T2) 29.33–35.44, and (T3) 35.48–53.23. Ascending tertiles of SAA reported lower ASPECT score (*p* < 0.001), greater Infarct volume (*p* < 0.001), greater CRP (*p* < 0.001), greater NIHSS scores during hospitalization (*p* < 0.001), greater three-month NIHSS scores (*p =* 0.024), lower three-month BI scores (*p* < 0.001), greater proportions of basal ganglia lesion (*p* < 0.001), and greater WBC (*p* = 0.002) (Table 2). The unfavorable and favorable result groups presented remarkable differences for the SAA (χ2 = 160.76, *p* < 0.001). For the unfavorable result group, the percentage of patients in the minimal tertile (2.87–28.84) and the maximum tertile (37.32–51.98) were notably lower and greater, separately. Besides, the number of patients with unfavorable results after AIS was 5 (4.13%), 23 (19.01%), and 93 (76.86%) in T1, T2, and T3, separately (Table 3).

**Table 2:** Baseline characteristics exhibited by patients with ICH according to SAA tertiles.

Variables	SAA			*p* value
	Q1 (≤ 29.23, *n* = 135)	Q2 (29.33–35.44, *n* = 135)	Q3 (≥ 35.48, *n* = 135)	
**Demographic characteristics**				
Gender, female, *n* (%)	69 (51.11)	72 (53.33)	83 (61.48)	0.196
Age, years, mean ± SD	68 (63–71)	67 (59–71)	68 (63–72)	0.518
Education years, medium value (IQR)	4 (3–7)	6 (3–8)	5 (3–7)	0.138
Married, *n* (%)	116 (85.93)	120 (88.89)	120 (88.89)	0.690
Vascular risk factors (%)				
Hypertension	90 (66.67)	95 (70.37)	102 (75.56)	0.272
Diabetes mellitus	59 (43.70)	62 (45.93)	69 (51.11)	0.457
Coronary heart disease	19 (16.05)	24 (11.64)	23 (12.33)	0.684
Atrial fibrillation	15 (11.11)	20 (14.81)	18 (13.33)	0.662
current smoking	48 (38.77)	55 (27.40)	54 (31.51)	0.639
Alcohol consumption	41 (30.37)	44 (35.62)	38 (32.88)	0.730
ONT, min, mean ± SD	160.85 ± 45.26	165.49 ± 44.79	161.45 ± 52.16	0.574
DNT, min, mean ± SD	69.31 ± 25.38	68.98 ± 23.10	65.52 ± 21.72	0.408
ASPECT, medium value (IQR)	10 (10–10)	10 (9–10)	9 (9–10)	<0.001
Infarct volume, ml, medium value (IQR)	2.22 (0.48–7.89)	7.96 (1.12–20.80)	14.23 (2.03–42.12)	<0.001
**Laboratory findings (IQR)**				
Plt, ×10^9^/L, medium value (IQR)	210 (182–234)	217 (183–241)	219 (155–262)	0.592
Serum amyloid A, mg/L, medium value (IQR)	26.99 (22.65–29.33)	33.23 (32.11–34.67)	39.63 (37.55–45.23)	<0.001
WBC, ×10^9^/L, medium value (IQR)	5.96 (5.19–6.97)	5.97 (5.25–6.88)	6.62 (5.32–8.42)	0.002
CRP, mg/L, medium value (IQR)	6.24 (5.44–7.28)	6.36 (5.43–7.50)	7.38 (6.42–8.53)	<0.001
EMR, medium value (IQR)	0.44 (0.20–0.71)	0.42 (0.25–0.66)	0.48 (0.23–0.80)	0.445
PLR, medium value (IQR)	141.74 (104.73–179.35)	146.39 (110.25–185.35)	134.39 (98.39–176.0)	0.483
NLR, medium value (IQR)	2.85 (2.26–3.65)	2.94 (2.21–3.76)	3.12 (2.26–4.04)	0.203
Glucose, mmol/L, medium value (IQR)	5.2 (4.70–6.40)	5.40 (4.70–7.10)	5.30 (4.70–6.80)	0.390
TG, mmol/L, medium value (IQR)	1.5 (1.05–2.02)	1.36 (0.91–2.04)	1.22 (0.91–1.75)	0.054
TC, mmol/L, medium value (IQR)	4.53 (3.77–5.57)	4.58 (3.86–5.52)	4.35 (3.60–5.19)	0.291
HDL-C, mmol/L, medium value (IQR)	0.96 (0.81–1.21)	1.04 (0.89–1.20)	1.05 (0.84–1.27)	0.239
LDL-C, mmol/L, medium value (IQR)	2.54 (1.69–3.24)	2.57 (2.04–3.18)	2.51 (1.93–3.12)	0.424
ApoA, g/L, medium value (IQR)	1.28 (1.09–1.44)	1.25 (1.10–1.42)	1.27 (1.11–1.45)	0.803
ApoB, g/L, medium value (IQR)	0.88 (0.69–1.04)	0.85 (0.70–1.02)	0.85 (0.68–1.03)	0.835
Hcy, μmol/L, medium value (IQR)	12.55 (10.41–16.30)	12.12 (10.02–15.31)	13.35 (10.15–16.80)	0.189
Lesion location, *n* (%)				
Frontal lobe	21 (15.56)	23 (17.04)	24 (17.78)	0.884
Parietal lobe	15 (11.11)	18 (13.33)	16 (11.85)	0.850
Temporal lobe	35 (25.93)	37 (27.41)	42 (31.11)	0.621
Occipital lobe	16 (11.85)	19 (14.07)	20 (14.81)	0.761
Basal ganglia	47 (34.81)	65 (48.15)	84 (62.22)	<0.001
Brainstem	11 (8.15)	9 (6.67)	11 (8.15)	0.870
Cerebellum	14 (11.36)	16 (11.85)	16 (11.85)	0.907
**TOAST subtype (*n*.%)**				
Large artery atherosclerosis	48 (39.51)	49 (33.88)	63 (41.90)	0.113
Cardioembolism	30 (28.64)	38 (39.67)	48 (23.94)	0.052
Small vessel occlusion	20 (14.81)	23 (17.04)	26 (19.26)	0.624
Other or undetermined	19 (14.07)	21 (15.56)	20 (14.81)	0.943
**Neuropsychological function**				
NIHSS score during hospitalization, medium value (IQR)	4 (4–4)	6 (5–7)	10 (8–12)	<0.001
Three-month NIHSS score, medium value (IQR)	1 (1–2)	3 (2–4)	6 (5–8)	<0.001
MMSE score, medium value (IQR)	23 (18–26)	22 (17–25)	23 (17–26)	0.262
Three-month BI score, medium value (IQR)	90 (70–90)	80 (65–90)	60 (40–75)	<0.001
Three-month mRS score, medium value (IQR)	2 (1–2)	2 (2–2)	3 (2–4)	<0.001

*** *p* < 0.001.

**Table 3:** SAA tertiles of patients.

Variables	Unfavorable result (*n* = 121)	Favorable result (*n* = 284)	χ2	*p* value
SAA			160.76	<0.001
Tertile1 (2.45–29.23)	5 (4.13%)	116 (40.85%)	54.59	<0.001
Tertile2 (29.33–35.44)	23 (19.01%)	98 (34.51%)	9.729	0.002
Tertile3 (35.48–53.23)	93 (76.86%)	28 (9.86%)	181.81	<0.001

*** *p* < 0.001.

### 3.3. Relationship between the level of SAA and unfavorable results after AIS

**Figure 3:**
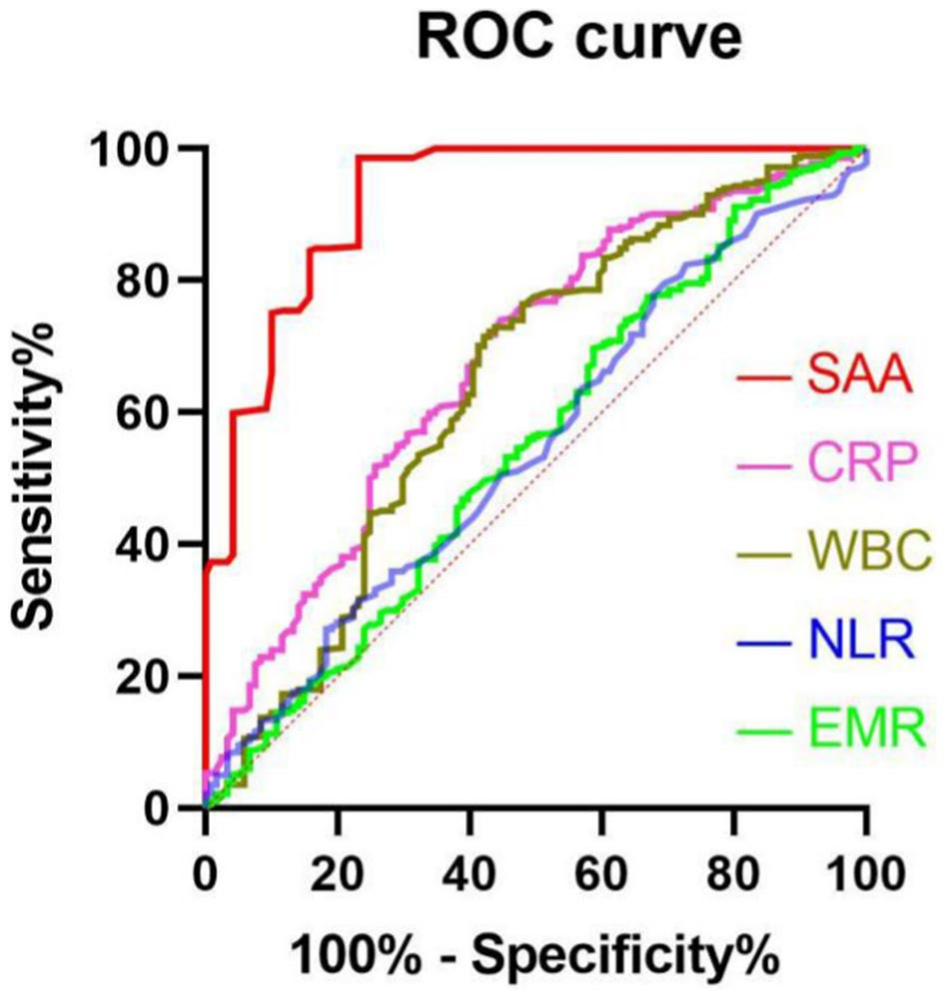
The ROC curves in terms of predicting unfavorable results. Predicted values of WBC, EMR, NLR, CRP, and SAA for unfavorable results in AIS patients at 3 months after undergoing IVT therapy. AUC 0.925 (95%CI, 0.895–0.954; *p*<0.001) in terms of SAA; AUC 0.677 (95%CI, 0.619–0.735; *p*<0.001) in terms of CRP; 0.643 (95%CI, 0.581–0.706; *p*<0.001) for WBC; 0.549 (95%CI, 0.486–0.612, *p =* 0.116) in terms of NLR; 0.547 (95%CI, 0.486–0.608; *p =* 0.136) in terms of EMR. SAA had a COV of 37.39, with specificity and sensitivity of 77.23% and 84.45%, separately.

We performed multivariate logistic regression study by considering small vessel occlusion, cardioembolism, basal ganglia, three-month BI score, NIHSS score during hospitalization, three-month NIHSS score, ASPECT score, infarct volume, EMR, NLR, WBC, CRP, and SAA as independent variables and confirmed that NIHSS score during hospitalization (OR 2.112, 95% CI: 1.895–2.688, *p* = 0.043) and three-month NIHSS score (OR 2.037, 95% CI: 1.341–2.971, *p* = 0.016), infarct volume (OR 1.452, 95% CI: 1.023–1.987, *p* = 0.039), and SAA (OR 2.874, 95% CI: 1.764–4.321, *p* < 0.001) independently predicted unfavorable results at 3 months after AIS (Table 4). There was a positive correlation between levels of SAA and the CRP (*r* = 0.367, *p* < 0.001). Furthermore, correlation analysis demonstrated that the levels of SAA were weak and positively associated with the NLR in all patients (*r* = 0.140, *p* = 0.005), and SAA was also positively associated with WBC in all patients (*r* = 0.216, *p* < 0.001). There was a negative correlation between levels of SAA and the EMR (*r* = −0.120, *p* = 0.016). The greater SAA levels during hospitalization corresponded to the greater mRS score at 3 months (*r* = 0.693, *p* < 0.001). The greater SAA levels during hospitalization also corresponded to the greater three-month NIHSS score (*r* = 0.763, *p* < 0.001). We considered all patients in logistic regression models without any adjustment and models with multiple adjustments, using unfavorable results and minimal tertile as dependent and reference variables in terms of the analysis of SAA, separately (Table 5). In the logistic regression model without regulations, in terms of cases with admission SAA, the maximum quartile exhibited more unfavorable results in terms of the minimal tertile (non-regulated: OR 5.327, 95% CI: 2.547–11.198, *p* < 0.001). When the regulation was completed in terms of marital status, education years, sex, and age (Model 1b), the odds ratio (OR) of the subjects in the maximum was 5.269 (95% CI 2.389–10.765, *p* < 0.001) for unfavorable results in comparison with the subjects in the minimal tertile. In the logistic regression model that regulated for the confounders of marital status, education years, sex, age, vascular risk factors (alcohol consumption, current smoking, atrial fibrillation, coronary heart disease, diabetes mellitus, and hypertension), the maximum tertile of SAA exhibited the capability of carrying out an independent prediction of unfavorable results after AIS (Model 2c: OR = 5.165, 95% CI = 2.395–11.594, *p* < 0.001). When all confounders revealed in the logistic regression model (Model 3d) were regulated, the correlation of SAA and stroke result continued to be significant at the maximum with OR of 4.127 (95% CI: 1.695–10.464, *p* = 0.001). The best COV of the SAA with the optimal discriminated unfavorable results was 37.39 (77.23% sensitivity and 84.45% specificity). SAA had a greater accuracy rate when compared with WBC (AUC 0.925 versus 0.643) and CRP (AUC 0.925 versus 0.677; *p* < 0.001) (Figure 3).

**Table 4:** Multivariate logistic regression analysis of three-month unfavorable results after AIS.

Variables	OR	95% CI	*p*
Small vessel occlusion	1.124	0.909–1.345	0.053
Cardioembolism	1.443	1.212–1.988	0.054
Basal ganglia	1.537	0.967–1.653	0.064
Three-month BI score	1.377	0.724–2.617	0.329
NIHSS score during hospitalization	2.112	1.895–2.688	0.043
Three-month NIHSS score	2.037	1.341–2.971	0.016
ASPECT score	0.984	0.569–1.456	0.762
Infarct volume	1.452	1.023–1.987	0.039
SAA	2.874	1.764–4.321	<0.001
EMR	0.986	0.543–1.245	0.875
NLR	0.853	0.474–1.828	0.059
WBC	1.124	0.942–2.187	0.064
CRP	1.025	0.839–1.364	0.717

*** *p* < 0.001.

**Table 5:** Non-regulated and regulated relationships of quartile of SAA levels and unfavorable results at 90 days.

	Tertile	OR^a^	95% CI	*p* value
Non-regulated	Middle	4.865	2.143–8.647	<0.001
	Maximum	5.327	2.547–11.198	<0.001
Model 1^a^	Middle	4.328	2.065–8.721	<0.001
	Maximum	5.269	2.389–10.765	<0.001
Model 2^c^	Middle	4.664	2.175–8.546	<0.001
	Maximum	5.165	2.395–11.594	<0.001
Model 3^d^	Middle	4.439	1.595–11.782	0.014
	Maximum	4.127	1.695–10.464	0.032

*** *p* < 0.001. ^a^Reference OR (1.000) is the minimal tertile of RDW in terms of PSD. ^b^Model 1: regulated in terms of marital status, education years, sex, and age. ^c^Model 2: regulated in terms of covariates of Model 1, based on in-depth regulation in terms of Vascular risk factors. ^d^Model 3: regulated in terms of covariates of Model 2, based on in-depth regulation in terms of variables with *p* < 0.05 in univariate analysis (BI score, mRS score, baseline NIHSS score, basal ganglia hematoma, CRP, and WBC). A Reference OR (1.000) is the minimal tertile of SAA for unfavorable results after AIS. B Model 1: regulated in terms of age, sex. C Model 2: regulated in terms of covariates of Model 1 and based on in-depth regulation for Vascular risk factors (ONT, DNT, ASPECT, Infarct volume, Hypertension, diabetes mellitus, current smoking, and alcohol consumption). D Model 3: regulated in terms of covariates from Model 2 and further regulated for variables (*p* < 0.05) within univariate investigation (WBC, CRP, EMR, NLR, WBC, CRP, Small vessel occlusion, Cardioembolism, Basal ganglia, three-month BI score, ASPECT score, Infarct volume, NIHSS score during hospitalization, and three-month NIHSS score).

## 4. Discussion

This paper has been the initial prospective cohort study based on hospitals in China to expound the association between unfavorable prognosis and SAA at 3 months in AIS patients undergoing thrombolytic therapy. The major finding is elucidated in the following: (1) Serum SAA levels in AIS patients were positively associated with admission CRP, NLR, WBC, NIHISS score, and mRS score. (2) Serum SAA can independently predict worsening prognosis at 3 months. (3) Serum SAA levels were notably greater in unfavorable-prognosis AIS patients subjected to IVT therapy compared with favorable-prognosis AIS patients subjected to IVT therapy. (4) In comparison with CRP, NLR, WBC, and EMR, serum SAA levels were notably more discriminatory in terms of clinical results in accordance with the ROC curve. (5) SAA at high levels during hospitalization showed a significant relationship with the unfavorable result of AIS patients 3 months after undergoing IVT therapy and confirmed that when main confounders were regulated, patients of the maximum SAA quartile achieved an unfavorable result risk 4.127-fold over the risk of the minimal SAA tertile. The above results confirmed that SAA may be effective in terms of the prediction of unfavorable results in AIS patients 3 months after undergoing IVT therapy from an epidemiological perspective. Therefore, the findings demonstrate that the up-regulated serum SAA levels show a notable relationship with inflammation and a worsening prognosis in AIS patients after IVT.

IVT therapy, i.e., an effective strategy to restore blood flow in the ischemic brain, is currently one of the most effective treatments for ACI (32). Even elderly patients over 80 years old can still benefit from IVT treatment (33). Although considerable progress has been made, it is estimated that less than 10% of all AIS patients benefit from reperfusion strategies (e.g., IVT and embolectomy) (34). Inflammation is a major contributor to reperfusion injury and may cause hemorrhagic transformation of cerebral infarcts and lead to unfavorable prognosis or even death as a clinical result (35). The inflammatory response following AIS has also been demonstrated to be associated with secondary brain injury following infarct-induced primary brain injury (35). The inflammatory response could be activated within minutes of the stroke event and last for days to weeks, or even longer (36). SAA has been confirmed as one of the highly conserved acute phase proteins that play a certain role in the chemotactic recruitment of inflammatory cells. SAA can be expressed in acute and chronic inflammation, facilitate the chemotaxis and adhesion of monocytes/macrophages, increase the inflammatory infiltration of atherosclerotic plaques, and up-regulate the expression of various inflammatory factors and the activation of inflammation-associated signaling pathways (37–39). The concentration of SAA can rise rapidly when the body is infected, injured, or inflamed. The production of SAA in the acute phase is triggered by pro-inflammatory cytokines that include interleukin-1, interleukin-6, and transforming growth factor-β (40, 41). Furthermore, an experimental animal study revealed that the concentration of SAA was notably greater within cerebral ischemia mice, and this concentration had the capability of mediating microglia activation with the use of a gene knockout technique (42). Recently, as revealed by considerable evidence, SAA takes on critical significance in the inflammation of several human diseases (e.g., ischemic stroke receiving endovascular thrombectomy, aSAH, traumatic brain injury, and acute myocardial infarction) (23, 28, 43, 44). Consistent with SAA, CRP refers to an acute phase protein and a well-known marker of inflammation of which peripheral blood concentration indicates the brain’s inflammatory response degree and the systemic inflammatory response degree (45–47). Patients with AIS due to arterial occlusion suffer from a local acute inflammatory response and changes in inflammatory cytokine levels (48). As revealed by the result of Wnuk et al., elevated CRP levels are correlated with poor short-and long-term functional outcomes in AIS patients treated with IVT (49). Experimental animal studies have found that CRP levels after ischemic events correlate with infarct size and are a good marker for the assessment of AIS prognosis (50). Furthermore, higher CRP levels are also related to delayed cerebral ischemia and vasospasm after subarachnoid hemorrhage (51). The association between CRP and SAA that we found (*r* = 0.367, *p* < 0.001) is in agreement with previous research (52). The intriguing finding here was that serum SAA levels showed a close relationship with systemic inflammation, as revealed based on the levels of CRP. Therefore, it can be hypothesized that serum SAA may be involved, at least, in the severe systemic inflammation arising from AIS patients receiving IVT therapy.

Until now, it was still unknown if the SAA circulation showed a relationship to AIS prognosis after IVT. In this paper of 405 AIS patients after IVT, we showed that the concentration of serum SAA was strongly associated with the NIHISS scores which is a known determinant of the prognosis of AIS receiving IVT therapy (14–18). Only a few reports have investigated the prognostic roles of blood inflammatory markers for 90-day functional results in patients who received IVT. Chen et al. found that greater NLR was associated with unfavorable results at 3 months (53). Furthermore, as revealed by the result of Qu et al., high WBC counts and CRP levels after IVT showed a relationship to unfavorable functional results in 447 patients at 3 months (54). Chen et al. explored the prognostic roles of differential leukocyte counts in-depth. A lower EMR was independently associated with unfavorable results and dead status in AIS patients after IVT (17). Besides, serum SAA levels under the ROC curve exhibited notable accuracy of prognosis for distinguishing AIS patients who had an unfavorable prognosis from those who had a favorable prognosis within 3 months after receiving IVT therapy. SAA was found to be a strong predictor of unfavorable results (AUC: 0.925) in comparison with CRP (AUC: 0.677), NLR (AUC: 0.549), WBC (AUC: 0.643), and EMR (AUC: 0.547). In accordance with the AUC of SAA, SAA is capable of effectively predicting AIS prognosis when IVT therapy is completed, with the specificity and sensitivity of 84.45% and 77.23%, separately. In general, the increased levels of SAA may be reported as a high level of inflammation, which exhibits a greater probability of contributing to the prognosis of AIS patients receiving IVT therapy. In other words, it is understood that increased levels of SAA in AIS patients after IVT could certainly be a valuable indicator of an unfavorable functional prognosis.

## 5. Conclusion

In brief, the up-regulation of SAA levels in circulating blood shows a correlation with NIHISS and admission mRS scores besides NLR, and serum SAA levels show an independent relationship with unfavorable results 3 months after the diagnosis of AIS receiving IVT, as confirmed by the result of this study of 405 AIS patients subjected to IVT. Additional studies with large sample sizes and multiple centers are needed to fully investigate these associations. It is imperative to investigate the mechanism of association between serum SAA levels and the prognosis of AIS receiving IVT in-depth.

## Data availability statement

The original contributions presented in the study are included in the article/supplementary material, further inquiries can be directed to the corresponding author.

## Ethics statement

The studies involving human participants were reviewed and approved by the First Affiliated Hospital of Anhui University of Science and Technology (First People’s Hospital of Huainan) (approval number: KJ2019A0096). Participants provided informed consent prior to inclusion in this study.

## Author contributions

YL and QC designed the experiments, carried out the experiments, analyzed the experimental results, and wrote the manuscript. YL, QC, JH, MX, CY, TL, and XD collected the clinical information. MX performed language editing. QC, YL, MX, CY, JH, and XD contributed to editorial changes in the manuscript. All authors contributed to the article and approved the submitted version.

## Funding

This work was supported by the Natural Science Research Projects in Anhui Universities (approval number: KJ2019A0096) and the Project of Huainan City Science and Technology Plan (approval number: 2022161).

## Conflict of interest

The authors declare that the research was conducted in the absence of any commercial or financial relationships that could be construed as a potential conflict of interest.

## Publisher’s note

All claims expressed in this article are solely those of the authors and do not necessarily represent those of their affiliated organizations, or those of the publisher, the editors and the reviewers. Any product that may be evaluated in this article, or claim that may be made by its manufacturer, is not guaranteed or endorsed by the publisher.
